# Relationship of Complete Blood Count Derived Biomarkers With Methotrexate Resistance

**DOI:** 10.7759/cureus.50765

**Published:** 2023-12-19

**Authors:** Tinatin Chikovani, Nestan Gvetadze, Luka Abashishvili, Levan Shalamberidze, Nino Kikodze

**Affiliations:** 1 Immunology, Tbilisi State Medical University, Tbilisi, GEO; 2 Molecular and Medical Genetics, Tbilisi State Medical University, Tbilisi, GEO; 3 Rheumatology, V. Tsitlanadze Scientific-Practical Center of Rheumatology, Tbilisi, GEO

**Keywords:** plr, das28, methotrexate resistance, cbc derived biomarkers, rheumatoid arthritis

## Abstract

Background

Rheumatoid arthritis (RA) is an autoimmune disease characterized by chronic inflammation and joint damage. Among the therapeutic agents, methotrexate remains a cornerstone of initial treatment. Complete blood count (CBC)-derived biomarkers such as neutrophil-to-lymphocyte ratio (NLR), platelet-to-lymphocyte ratio (PLR), monocyte-to-lymphocyte ratio (MLR), systemic immune-inflammation index (SII), and systemic immune response index (SIRI) have been extensively studied in various diseases. Still, their specific role in RA patients undergoing methotrexate treatment has not been investigated.

Objective

This study aimed to investigate the relationship of CBC-derived biomarkers with methotrexate resistance in newly diagnosed rheumatoid arthritis patients.

Methods

We performed a comprehensive analysis of 54 RA patients, divided into methotrexate-resistant (MTXR) and methotrexate-sensitive (MTXS) groups. Analysis of variance (ANOVA) was used to assess differences in hematological biomarkers between groups. Standard t-tests were used to compare specific biomarkers between the MTXR and MTXS groups. The chi-squared test was used to compare categorical variables between groups. Pearson's correlation test was also used to examine correlations between these biomarkers and Disease Activity Score 28 (DAS28) in both groups. Receiver operating characteristic (ROC) curve analysis was performed for each biomarker to determine predictive ability.

Results

A statistically increased PLR ratio was observed in the MTXR group compared to the MTXS group. Significant correlations between DAS28 and NLR, PLR, SII, and SIRI were observed in the MTXR group. In contrast, these correlations were absent in the MTXS group. In addition to PLR, DAS28 and ESR were significantly higher in the MTXR group than in the MTXS group. None of these biomarkers showed prognostic value for methotrexate treatment outcomes.

Conclusion

PLR could be used as a biomarker for resistance to methotrexate treatment in a specific RA patient population. Increased PLR and ESR, together with higher DAS28, might be associated with a more pronounced inflammatory state in MTXR patients.

## Introduction

Rheumatoid arthritis (RA) is a chronic autoimmune disease that causes joint damage and is increasingly associated with vascular, metabolic, bone, and psychiatric comorbidities. RA affects up to 1% of the population, mostly women, and can manifest at any age or stage of life [1], with genetic, epigenetic, and environmental variables contributing to the development of the disease [2].

The pathophysiology of RA is characterized by a complex interplay of many cells, including leukocytes, synovial fibroblasts, chondrocytes, and osteoclasts, resulting in a breakdown of immunological homeostasis [3]. Due to their propensity to generate degradative enzymes and reactive oxygen species, neutrophils have the highest cytotoxic potential of any cell involved in the pathophysiology of RA and are even a source of providing the autoantigens that drive the mechanism of the autoimmune process [4].

Considering that inflammation is a significant predictor and fundamental underlying mechanism of RA, its assessment with accurate indicators is crucial for predicting the long-term fate of a patient. In daily practice, the most commonly used markers for this purpose are erythrocyte sedimentation rate (ESR) and C-reactive protein (CRP) [5].

Non-invasive and cost-effective biomarkers derived from the complete blood count (CBC), such as neutrophil-lymphocyte ratio (NLR), monocyte-lymphocyte ratio (MLR), platelet-lymphocyte ratio (PLR), systemic immune-inflammation index (SII), and systemic immune response index (SIRI), have received increasing attention in recent years as potential prognostic tools in various inflammatory and autoimmune diseases [6-10]. According to recent studies, NLR and PLR may be strongly associated with the underlying pathogenetic pathways of RA, as well as with disease development and severity [11-13]. PLR may be an underlying sign of persistent subclinical inflammation in RA patients [14]. SII has been studied alongside NLR and PLR to aid in the diagnosis and prediction of disease activity in rheumatoid arthritis [15]. Likewise, in a recent study, SIRI demonstrated its potential to be used as a novel biomarker to aid in the diagnostic process, demonstrate RA disease activity, and predict RA-associated interstitial lung disease (RA-ILD) and tumor development [16]. According to another study, MLR may have moderate accuracy as a complementary diagnostic indicator for RA diagnosis in patients with undifferentiated inflammatory arthritis [17].

Although the treatment of RA has evolved significantly over the years, with various therapeutic strategies aimed at alleviating symptoms, preventing joint damage, and improving the overall quality of life for patients, MTX, a cornerstone of RA treatment, remains the first-line initial therapy for newly diagnosed RA patients. While it has demonstrated efficacy in controlling disease activity and reducing joint destruction, individual responses to MTX therapy can vary widely, highlighting the need for reliable prognostic indicators to guide treatment decisions. Although the aforementioned biomarkers have been extensively studied in various medical contexts, a notable research gap exists in the study of these biomarkers about methotrexate resistance.

In the present study, we therefore aimed to investigate the relationship of NLR, MLR, PLR, SIRI, and SII with MTX resistance in RA patients.

## Materials and methods

Study population

This retrospective study included 54 newly diagnosed patients with rheumatoid arthritis from the V. Tsitlanadze Scientific-Practical Center of Rheumatology in Tbilisi, Georgia. Patients were eligible for inclusion if they met the diagnostic criteria for rheumatoid arthritis according to the criteria established by the European Alliance of Associations for Rheumatology (EULAR) and the American College of Rheumatology (ACR).

Each patient's demographic data and detailed medical history were collected. The clinical and laboratory evaluation included recording the number of swollen and tender joints (SJC and TJC), erythrocyte sedimentation rate (ESR), C-reactive protein (CRP), rheumatoid factor (RF), anti-CCP antibodies, complete blood count, and disease activity assessment using the DAS28 score. DAS28 has been calculated using the following parameters: SJC, TJC, and ESR. Patients were required to exhibit high disease activity (DAS28>3,2).

The control group from the same V. Tsitlanadze Scientific-Practical Center of Rheumatology consisted of 28 age- and sex-matched subjects without any type of cancer, acute or chronic infections, or autoimmune diseases.

Exclusion criteria

Patients with systemic diseases such as diabetes mellitus, hypertension, chronic renal failure, coronary artery disease, chronic obstructive pulmonary disease, malignancy, acute or chronic infection, pregnancy or postpartum, granulomatous disease, or any inflammatory disorder were excluded from the study.

All patients were started on methotrexate, which was administered according to standard clinical protocols, up to 25 mg weekly.

Data collection

Complete blood count (CBC) data were collected from all patients. CBC measurements included neutrophil, lymphocyte, monocyte, and platelet counts, which allowed the calculation of NLR, PLR, MRL, SIRI, and SII. NLR, PLR, and MLR were calculated as the absolute neutrophil/platelet/monocyte count divided by the absolute lymphocyte count.

The systemic immune inflammation index (SII) was calculated as peripheral platelet count × neutrophil/lymphocyte count. SIRI was calculated as neutrophil count × monocyte/lymphocyte count.

Response assessment

After a three-month treatment period with methotrexate, patients were assessed for treatment response. Based on the evaluation of disease activity markers (DAS28), improvement or disappearance of clinical symptoms, reduction of tender and swollen joint counts, decrease of acute phase reactants (ESR and CRP), and improvement in performing vocational and avocational activities, patients were categorized into two groups: responders (methotrexate sensitive) (MTXS) and non-responders (methotrexate resistant) (MTXR). MTXS patients who achieved remission or a significant reduction in disease activity continued their MTX treatment. MTXR patients who did not achieve remission or showed inadequate improvement in disease activity were switched to treatment with tocilizumab, an interleukin-6 receptor inhibitor.

Statistical analysis

Statistical analyses were performed using Prism 9 and IBM SPSS Statistics for Windows, Version 26 (released 2010; IBM Corp., Armonk, New York, United States). Descriptive statistics were used to summarize patient demographics and baseline characteristics. All quantitative data were expressed as mean +/- standard deviation. Unpaired t-tests and analysis of variance (ANOVA) were used for group comparisons where appropriate. The statistical significance between groups for categorical variables was calculated using the chi-squared test. Receiver operating characteristic (ROC) curve analysis was performed to determine the optimal cut-off values for NLR, PLR, SII, and SIRI in predicting treatment response. Pearson's correlation test was used for the correlation study. All tests were two-tailed. Differences below 0.05 were considered statistically significant.

Ethical considerations

The study protocol was approved by the Tbilisi State Medical University Biomedical Research Ethics Committee (approval number N1-2022/94). All participants provided informed consent prior to enrollment.

## Results

Of the 54 RA patients, 48 (89%) were female and 6 (11%) were male.

There were 37 patients in the MTXS group, 17 patients in the MTXR group, and 28 age- and sex-matched patients in the control group. The mean age and sex distribution of patients in the MTXS, MTXR, and control groups were not significantly different.

ANOVA analysis showed statistically significant differences between MTXS, MTXR, and control groups in NLR (p=0.0159), PLR (p=0.0003), SII (p=0.0005), and SIRI (p<0.0001). There was no statistically significant difference between the groups for MLR. The ANOVA test also showed statistically significant differences between the groups for neutrophils (p<0.0001), lymphocytes (p=0.0025), monocytes (p=0.0023), and platelets (p<0.0001) (Table 1).

**Table 1:** Comparison between the study populations’ baseline clinical and serological characteristics SD: standard deviation; DAS28: disease activity score for 28 joints; ANA: antinuclear antibody; anti-CCP: anti-cycling citrullinated peptide antibody; RF: rheumatoid factor; CRP: C reactive protein; ESR: erythrocyte sedimentation rate; SII: systemic inflammatory index; SIRI: system inflammation response index; NLR: neutrophil-lymphocyte ratio; MLR: monocyte-lymphocyte ratio; PLR: platelet-lymphocyte ratio p1: MTXS group vs. MTXR group; p2: MTXS group vs. MTXR group vs. control group; p3: overall RA patients vs. control group P < 0.05 was considered statistically significant

	RA patients N=54	MTXS group n=37	MTXR group n =17	Control n=28	p^1^	p^2^	p^3^
Age (years), (mean ± SD)	52.70 ±13.68	52.24 ± 15.03	53.71 ± 10.50	48.29 ± 15.68	0.7190	0.5168	0.1912
Female, n (%)	48 (88.89%)	33 (89.19%)	13 (76.47%)	20 (71.42%)	0.2217	0.1807	0.0643
DAS28	5.88 ± 0.64	5.76± 0.64	6.14 ±0.57	N/A	0.0418	_	_
ANA positive (>1:80), n (%)	5 (9.3%)	1 (2.7%)	4 (23.53%)	0 (0 %)	0.0142	0.0031	<0.0001
RF, n (%)	33 (61.11%)	16 (43.24%)	17 (100%)	0 (0 %)	<0.0001	<0.0001	0.0001
Anti CCP, n (%)	43 (79.62%)	27 (72.97%)	16 (94.1%)	0 (0 %)	0.0732	0.0039	0.0103
CRP (mg/L), (mean ± SD)	28.86 ± 21.56	30.28 ± 20.37	25.76 ± 24.33	_	0.4805	_	_
ESR (mm/h) (mean ± SD)	34.28 ± 17.95	30.59 ± 17.73	42.29 ± 16.15	_	0.0247	_	_
Neutrophils (10^3 ^cells/mL)	5.53 ±1.74	5.91 ±1.84	4.69 ±1.17	3.65 ± 0.93	0.0159	<0.0001	<0.0001
Lymphocytes (10^3 ^cells/mL)	2.37 ± 0.88	2.57 ± 0.93	1.94 ± 0.55	2.05 ± 0.40	0.0121	0.0025	<0.0001
Monocytes (10^3 ^cells/mL)	0.56 ± 0.30	0.63 ± 0.32	0.39 ± 0.12	0.51 ± 0.12	0.0044	0.0023	<0.0001
Platelets (10^9^ cells /mL)	322.5 ±85.52	313.22 ± 92.67	342.65 ± 65.39	248.93 ± 43.93	<0.0001	<0.0001	<0.0001
NLR, (mean ± SD)	2.64 ± 1.38	2.58 ± 1.26	2.78± 1.63	1.85 ± 0.59	0.6114	0.0159	<0.0001
MLR, (mean ± SD)	0.26 ± 0.15	0.28 ± 0.16	0.23 ± 0.12	0.25 ± 0.06	0.2718	0.4099	<0.0001
PLR, (mean ± SD)	156.50 ±75.54	136.86 ± 57.83	199.39 ± 92.37	125.50 ± 30.23	0.0037	0.0003	<0.0001
SII, (mean ± SD)	861.7 ± 517.3	808.29 ± 443.10	977.85 ± 650.93	466.00 ± 207.82	0.2673	0.0005	<0.0001
SIRI, (mean ± SD)	1.51 ±1.10	1.70 ± 1.18	1.13 ± 0.82	63.30 ± 21.08	0.0815	<0.0001	<0.0001

Of the biomarkers of interest, only PLR showed a statistically significant difference between the MTXS and MTXR groups (p=0.0037). It was significantly higher in the MTXR group (Table 1). In addition to PLR, DAS28 (p=0.0418) and ESR (p=0.0247) were significantly higher in the MTXR group than in the MTXS group (Table 1).

Table 1 summarizes the demographic, clinical, and laboratory data of the subjects studied.

All investigated CBC-derived biomarkers, except MLR, significantly correlated with DAS28 in the MTXR group (for NLR, PLR, SII, and SIRI, p=0.0102, 0.0267, 0.0040, and 0.0121, respectively) (Table 2). In contrast, no associations were found between DAS28 and NLR, PLR, MLR, SII, and SIRI in the MTXS group (Table 2).

**Table 2:** Correlation between DAS-28 and CBC-derived biomarkers in MTXR and MTXS group DAS28: disease activity score for 28 joints; SII: systemic inflammatory index; SIRI: system inflammation response index; NLR: neutrophil-lymphocyte ratio; MLR: monocyte-lymphocyte ratio; PLR: platelet-lymphocyte ratio P < 0.05 was considered statistically significant

	DAS 28 versus									
	MTXR					MTXS				
	NLR	MLR	PLR	SII	SIRI	NLR	MLR	PLR	SII	SIRI
Pearson correlation coefficient (r)	0.54	0.60	0.45	0.66	0.59	-0.08	-0.04	0.09	-0.13	-0.18
P value	0.03^*^	0.07	0.01^*^	0.00^*^	0.01^*^	0.65	0.81	0.60	0.46	0.30

Prognostic evaluation of hematological biomarkers

The prognostic potential of NLR, PLR, SII, and SIRI in predicting treatment outcomes in RA patients receiving methotrexate was assessed using receiver operating characteristic (ROC) curve analysis.

**Figure 1:**
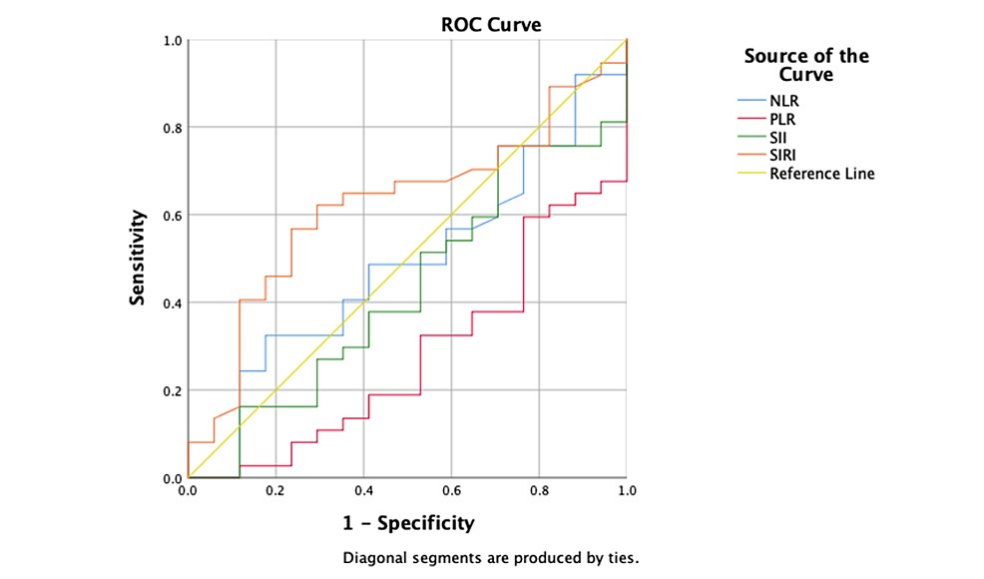
ROC curve analyzes the prognostic role of inflammatory biomarkers in RA patients under MTX treatment SII: systemic inflammatory index; SIRI: system inflammation response index; NLR: neutrophil-lymphocyte ratio; MLR: monocyte-lymphocyte ratio; PLR: platelet-lymphocyte ratio; ROC: receiver operating characteristic

ROC curve analysis showed that the area under the curve (AUC) for each biomarker did not exceed the threshold, indicating predictive ability. The AUC values for NLR, PLR, SII, and SIRI were 0.483, 0.277, 0.428, and 0.622 (Table 3). Table 3 and Figure 1 display the results of the ROC curve analysis for inflammatory biomarkers.

**Table 3:** ROC analysis results of CBC-derived biomarkers SII: systemic inflammatory index; SIRI: system inflammation response index; NLR: neutrophil-lymphocyte ratio; MLR: monocyte-lymphocyte ratio; PLR: platelet-lymphocyte ratio; ROC: receiver operating characteristic

Test result variable(s)	Area	Std. error^a^	Asymptotic sig.^b^	Asymptotic 95% confidence interval	
				Lower bound	Upper bound
NLR	0.483	0.083	0.845	0.320	0.647
PLR	0.277	0.072	0.009	0.135	0.418
SII	0.429	0.083	0.407	0.266	0.592
SIRI	0.622	0.079	0.152	0.467	0.778

## Discussion

The search for reliable biomarkers of treatment response in patients undergoing methotrexate therapy is one of the current areas of research. However, our understanding of its efficacy, particularly about specific inflammatory biomarkers, remains limited. Evaluating the role of hematological biomarkers, such as NLR, PLR, MLR, SII, and SIRI, in RA patients treated with methotrexate could provide remarkable insights into the complexities of disease management. The primary and potentially landmark finding of our study, which requires careful consideration, is the identification of a statistically significant difference in PLR ratios between the MTXR and MTXS groups. In the MTXR group, we observed a higher PLR compared to the MTXS group (p<0.0037). This may indicate a possible association between the PLR ratio and resistance to methotrexate treatment. We also calculated Pearson's correlation coefficients for these subgroups to explore possible associations between hematological biomarkers and disease activity. Notably, we found a statistically significant positive correlation between DAS28 and PLR, NLR, SII, and SIRI in the MTXR group but not in the MTXS group (p<0.0267, p<0.0102, p<0.0040, p<0.0121, respectively). Similar associations have been suggested in several studies where NLR, PLR, and SII were positively correlated with DAS28 [13,14,18,19] in RA patients.

The higher PLR levels in the MTXR group compared to the MTXS group, together with a statistically significant positive correlation between PLR and DAS28 in the MTXR group, raises several important considerations. Firstly, the higher PLR in the MTXR group compared to the MTXS group may indicate a more pronounced inflammatory state in these patients, possibly contributing to their resistance to methotrexate treatment. In addition, the observed difference in PLR may reflect different degrees of treatment response in RA patients. It suggests that MTXR individuals may have a distinct inflammatory profile that may be less responsive to MTX monotherapy. This finding raises interesting questions about the potential utility of PLR as a specific biomarker in a subset of RA patients who exhibit resistance to MTX therapy. While this correlation requires further validation in larger cohorts and prospective studies, it introduces the potential of PLR as a marker to identify patients who may benefit from alternative treatment strategies when faced with MTX resistance and once again underscores the importance of tailored treatment strategies for specific subsets of RA patients.

Despite extensive research into the usefulness of NLR, PLR, MLR, SII, and SIRI in a variety of inflammatory and autoimmune disorders [20,21,22,23], their prognostic ability to predict the potential outcome of methotrexate treatment has not been evaluated. Our results showed that, in the context of our RA study cohort, none of these hematological biomarkers showed the necessary discriminatory power to serve as effective prognostic tools. The AUC values for these biomarkers remained close to or below the threshold of 0.5, indicating results no better than chance. Similar to our findings, SII, NLR, and PLR were not considered to have predictive potential for treatment response in patients treated with Janus kinase inhibitors [24]. Conversely, in another study, NLR was found to be an independent predictor of subsequent treatment failure in newly diagnosed RA patients starting triple therapy with methotrexate, sulfasalazine, and hydroxychloroquine [25]. These contrasting findings suggest that the prognostic value of these CBC-derived biomarkers may be context-specific and dependent on the therapeutic regimen used, highlighting the need for further investigation and validation of their utility.

The limitations of our study include its retrospective nature, sample size, and the fact that data collection was performed in a single medical center. Future investigations should focus on replicating and extending this result, finding clear explanations for the correlation between PLR and DAS28 in the MTXR group, and evaluating the clinical implications of using PLR as a potential biomarker to guide treatment decisions in methotrexate-resistant RA patients.

## Conclusions

We found an association between an elevated PLR and resistance to methotrexate in RA patients. Thus, being an inexpensive, easily measured parameter, PLR could serve as a potential biomarker for detecting RA treatment outcomes. NLR, PLR, SII, and SIRI positively correlate with DAS28 only in the MTXR group of patients. Furthermore, patients with elevated PLR and ESR, alongside higher DAS28 scores, may indicate a more profound inflammatory state among MTXR patients. Our study did not reveal the effectiveness of NLR, PLR, SII, or SIRI as prognostic indicators for RA patients undergoing MTX therapy.
